# Morphine Induces Bacterial Translocation in Mice by Compromising Intestinal Barrier Function in a TLR-Dependent Manner

**DOI:** 10.1371/journal.pone.0054040

**Published:** 2013-01-18

**Authors:** Jingjing Meng, Haidong Yu, Jing Ma, Jinghua Wang, Santanu Banerjee, Rick Charboneau, Roderick A. Barke, Sabita Roy

**Affiliations:** 1 Department of Pharmacology, University of Minnesota Medical School, Minneapolis, Minnesota, United States of America; 2 Department of Surgery, Division of Infection, Inflammation, and Vascular Biology, University of Minnesota Medical School, Minneapolis, Minnesota, United States of America; 3 Department of Surgery, Veterans Affairs Medical Center, Minneapolis, Minnesota, United States of America; University of Nebraska Medical Center, United States of America

## Abstract

Opiates are among the most prescribed drugs for pain management. However, morphine use or abuse results in significant gut bacterial translocation and predisposes patients to serious infections with gut origin. The mechanism underlying this defect is still unknown. In this report, we investigated the mechanisms underlying compromised gut immune function and bacterial translocation following morphine treatment. We demonstrate significant bacterial translocation to mesenteric lymph node (MLN) and liver following morphine treatment in wild-type (WT) animals that was dramatically and significantly attenuated in Toll-like receptor (TLR2 and 4) knockout mice. We further observed significant disruption of tight junction protein organization only in the ileum but not in the colon of morphine treated WT animals. Inhibition of myosin light chain kinase (MLCK) blocked the effects of both morphine and TLR ligands, suggesting the role of MLCK in tight junction modulation by TLR. This study conclusively demonstrates that morphine induced gut epithelial barrier dysfunction and subsequent bacteria translocation are mediated by TLR signaling and thus TLRs can be exploited as potential therapeutic targets for alleviating infections and even sepsis in morphine-using or abusing populations.

## Introduction

Morphine is the most widely used analgesic worldwide for the management of pain. Morphine use is especially prevalent in patients undergoing invasive procedures that are associated with long operative times and extended hospitalization [1], [2]. Clinically, morphine use has been shown to be an independent risk factor for infection and infection-related morbidity in burn patients [3], [4]. Furthermore, clinical studies have reported that patients with sepsis, severe sepsis, and septic shock had significant higher circulating morphine levels than patients with systemic inflammatory response syndrome and healthy controls [5], while the opioid antagonist naltrexone has been shown to block acute endotoxic shock by inhibiting tumor necrosis factor-α production [6]. Studies using animal models show that both chronic morphine and morphine withdrawal can lower host defense to enteric bacteria such as *Salmonella enterica and Pseudomonas aeruginosa*, induce spontaneous sepsis in mice, and sensitize mice to mortality induced by *Acinetobacter baumannii* infection or lipopolysaccharide (LPS) [7]–[12]. In addition to bacterial translocation, morphine has been documented to increase serum IL-6 levels in rats and accelerate the progression of LPS-induced sepsis to septic shock [6], [13], [14]. Overall, both clinical and laboratory studies provide evidence that μ-opioid receptors are involved in the development and progression of various infectious diseases related to gut pathogens. However, the mechanisms underlying compromised gut immune function and increased susceptibility to infections after morphine treatment have not been well characterized. Therefore, the objective of the present study was to understand the correlation between morphine treatment and compromised gut barrier function, in order to support the development of novel strategies to treat or prevent gut bacterial infection in opioid-using or -abusing populations.

Epithelium is one of the most important components of intestinal mucosal immunity, which is required for prevention of potential pathogen invasion. The intestinal epithelium, as the first line of defense in the gut luminal environment, is not only a simple physical barrier but also plays an essential role in supporting nutrient and water transport and maintaining the homeostasis of the whole organism [15]. Not surprisingly, compromised barrier function allows the intestinal microbiota to translocate through the epithelium and leads to increased susceptibility to infection by gut pathogens, and faster progression of infectious disease. Gut epithelial cells play an important role in recognizing and preventing potential pathogen or antigen invasion. To accomplish these complicated functions, well-organized transmembrane and paracellular tight junction proteins are expressed in these polarized cells. Tight junction proteins in intestinal epithelium include transmembrane proteins such as occludin and claudin family members, which seal the paracellular pathway between the epithelial cells, as well as paracellular proteins such as zona occludens-1 (ZO-1) and zona occludens-2 (ZO-2), acting as scaffolding molecules. Disruption of gut tight junction barrier function has severe consequences including bacterial translocation from the gut leading to immune activation and inflammation [16].

Toll-like receptor (TLR) signaling is one of the most important components of innate immunity and has to be regulated tightly in gut epithelium to maintain the balance between normal and over-exuberant activation due to the presence of large amount of commensal bacteria in the lumen of the gastrointestinal tract [17]. Among all TLRs in the gut, TLR2 and TLR4 play important roles in physiological and pathological processes, and are both involved in intestinal permeability regulation. TLR2 and TLR4 have been shown to regulate the gate-keeping functions of the intestinal follicle-associated epithelium [18]. Paradoxically, activation of TLR4 by LPS increases intestinal monolayer permeability in a myosin light chain kinase (MLCK)-dependent manner [19], [20]. Meanwhile, there is evidence showing intracellular cross talk between MOR signaling and TLR signaling in various kinds of cells [3]. For example, morphine significantly inhibits tumor necrosis factor-α (TNF- α), but not interleukin-6 (IL-6) production, in a MOR-independent manner in polyglycan-stimulated peripheral blood mononuclear cells [21]. However, the intracellular mechanism underlying how morphine compromises epithelial barrier function via modulating TLRs is still not defined. In the present study, we hypothesize that morphine disrupts the barrier function of gut epithelium by increasing the sensitivity of gut epithelial cells to TLR activation, resulting in bacterial translocation from the gut lumen. We investigated the effects of morphine on gut barrier function in wild type (WT), TLR2 knockout, TLR4 knockout, and TLR2/4 double knockout mice. The direct effects of morphine on gut epithelial cells were further studied with rodent small intestinal and colonic epithelial cell lines, IEC-6 and CMT-93, respectively. Our results from *in vivo* and *in vitro* studies indicate that morphine treatment compromises gut barrier function in a TLR-dependent manner.

## Materials and Methods

### Experimental animals

Pathogen-free B6129PF2, C57BL/6J, B6.129^Tlr2tm1Kir^/J (TLR2 knockout) and C57BL/10ScNJ^-Tlr4lps-del^ (TLR4 knockout) mice were obtained from the Jackson Laboratory (Bar Harbor, ME). We crossed TLR2 knockout withTLR4 knockout mice to generate TLR2/4 double knockout mice. MOR knockout (MORKO) mice (C57BL/6129/Ola genetic background) were generated by Loh and colleagues [22]. Briefly, a XhoI/XbaIfragment, which spans exons 2 and 3, was replaced with a Neor cassette, followed by the ligation of a thymidinekinase expression cassette to the 3′ end of this segment. All animals were housed in a specific-pathogen-free facility under barrier conditions. All animal experiments were done in accordance with the Institutional Animal Care and Use Committee's guidelines at the University of Minnesota. The protocol was approved by Institutional Animal Care and Use Committee (IACUC) at the University of Minnesota (protocol# 0909A72719). All surgery was performed under isoflurane anesthesia, and all efforts were made to minimize suffering.

### Animal treatment

Mice received morphine and pellet implantation method as described [23]. Using this method, plasma levels of morphine are in the 0.6–2.0-microg/ml range (range seen in opioid abusers and patients on opioids for moderate to severe pain). Furthermore, this model is commonly used in the study of opiate dependence and addiction [23]. Briefly, placebo or 75 mg morphine pellets (National Institutes of Health [NIH]/National Institute on Drug Abuse [NIDA], Bethesda, MD) were inserted in a small pocket created by a small skin incision on the animal's dorsal side; incisions were closed using surgical wound clips (Stoelting, 9 mm Stainless Steel, Wooddale, IL). Animals were injected with MLCK inhibitor ML-7 (2 mg/kg) overnight before LPS or Lipoteichoic acid (LTA) treatment. At this dose, ML-7 successfully inhibited activity of myosin light chain kinase and protected the barrier function of endothelial cells in mice [24].

### Intestinal permeability

All animals were gavaged with ampicillin-resistant *E. coli* (2×10^7^ CFU suspended in 400 µl of sterile saline) or FITC-dextran (600 mg/kg body weight in 20 mg/ml concentration) utilizing a 4-cm long, curved needle with a plastic ball at the tip. After sacrifice, MLN and liver were collected and cultured on LB plates containing 100 µg/ml of ampicillin to measure bacterial translocation. Whole blood FITC-dextran concentration was determined by fluorometry based on a standard curve.

### Immunofluorescence

Sections of small intestinal and colonic tissue from all mice sacrificed for tight junction staining were frozen in TFM™ tissue freezing medium (TBS, Durham, NC). At least five sections from each of three animals for each condition were analyzed by immunofluorescence microscopy. Representative images are shown. For immunostaining, 5 µm frozen sections were fixed with 1% paraformaldehyde in PBS for 10 min at room temperature. After washing in PBS and blocking of nonspecific binding sites with 5% bovine serum albumin (BSA), tissues were incubated with polyclonal rabbit anti-occludin or rabbit anti-ZO-1 (both used at 5 µg/ml, Invitrogen) in PBS with 5% bovine serum albumin (BSA) for 120 min at room temperature. After washing, sections were incubated with rhodamine phalloidin (Invitrogen) and DyLightTM 488-conjugated AffiniPure Donkey anti-rabbit IgG (0.075 mg/ml, Jackson Lab, WestGrove, PA) for 60 min. Sections were then washed and mounted under coverslips using ProLong Gold antifade reagent with DAPI (Invitrogen). Sections were imaged using a confocal microscope (Nikon). Image J RG2B co-localization software was used to quantify the intensity of yellow fluorescence (indicating co-localization of green and red) and normalized to blue fluorescence (DAPI).

### Western blots

Cells were lysed with radioimmunoprecipitation assay (RIPA) buffer (Sigma). Lysates (80 µg protein per lane) were separated by SDS-PAGE, and proteins were electrotransferred from gel onto nitrocellulose membrane. Membranes were blocked in Tris-buffered saline, 0.1% Tween 20, 5% BLOT-QuickBlockerTM (G-Biosciences, St Louis, MO), and incubated with primary and secondary IRDye® anti-IgG Abs (LI-COR Biosciences). Protein bands were visualized using Odyssey infrared imaging system (LI-COR Biosciences).

### Realtime PCR

Total cellular RNA was extracted using TRIzol (Invitrogen), and cDNA was synthesized with the M-MLV Reverse Transcription Kit (Promega). Primers for TLR2, TLR4, and 18S ribosomal RNA were purchased from IDT. Quantitative real-time polymerase chain reaction (PCR) was performed on an Applied Biosystems 7500 Realtime PCR Detection system. All samples were run in triplicate, and relative mRNA expression levels were determined after normalizing all values to 18S RNA. Primer sequence: 18s 5′-GTAACCCGTTGAACCCCATT-3′;5′-CCATCCAATCGGTAGTAGCG-3′; TLR2 5′-CGCCTAAGAGCAGGATCAAC-3′; 5′-GGAGACTCTGGAAGCAGGTG-3′; TLR4 5′-CCAGAGCCGTTGGTGTATCT-3′; 5′-TCAAGGCTTTTCCATCCAAC-3′.

### Epithelial cell isolation

Epithelial cells were isolated as described previously [25]. Small intestines were excised from mice, flushed with HBSS/2% FBS, opened longitudinally, and cut into 0.5-cm pieces. The tissue was further washed and incubatedin HBSS/2% FBS, 0.5 mM EDTA, and 1 mM DTT, at 37°C in a shaking water bath for 45 min. The cell suspension released upon vigorous shaking was layered on a discontinuous 25%/40% Percoll gradient (Sigma) and centrifuged at 600×g for 10 min. Intestinal epithelial cells (IEC) were collected from the interphase and incubated with anti-cytokeratin antibody (BD Pharmingen), anti-TLR2 and anti-TLR4 antibodies (eBiosciences).

### Cell culture and treatment

IEC-6 and CMT-93 cell lines were purchased from American Type Culture Collection (Manassas, VA) and cultured as recommended by the supplier. IEC-6 and CMT-93 cells are rodent small intestinal and colonic epithelial cell lines, which have been used for studying intestinal barrier and integrity in several publications [26], [27]. Cells were pretreated with MLCK inhibitor ML-7 before LPS (1 µg/ml, Sigma) or LTA (5 µg/ml, Sigma) stimulation. Inactivation of MLCK by ML-7 has been shown to protect barrier function in various endothelial and epithelial cell lines [24], [28].

### Measurement of trans-epithelial resistance

ECIS 1600R (Applied BioPhysics, Troy, NY) was used to measure trans-epithelial resistance (TER) of epithelial monolayers as described previously [29]. Epithelial cells were seeded in the wells of the electrode array and grown to confluence as indicated below. Then medium was exchanged, and baseline TER was measured for 60 min to equilibrate monolayers. Afterward, 400 µl of medium containing ML-7 (10 µM), LPS (1 µg/ml), or LTA ((5 µg/ml) was applied to the wells.

### Statistical analysis

Experiment data were plotted and analyzed using GraphPad Prism (GraphPad Software, Inc.). Parametric data were compared using Student's t-test and nonparametric data using Mann–Whitney test. For multiple-group comparison, data were analyzed by ANOVA one-way analysis, followed by Bonferroni post-test. Quantitative data are expressed as means ± SE of three experiments. Points represent values of individual mice, and lines depict mean values.

## Results

### Chronic Morphine compromises the barrier function of gut epithelium and promotes bacterial translocation

**Figure 1:**
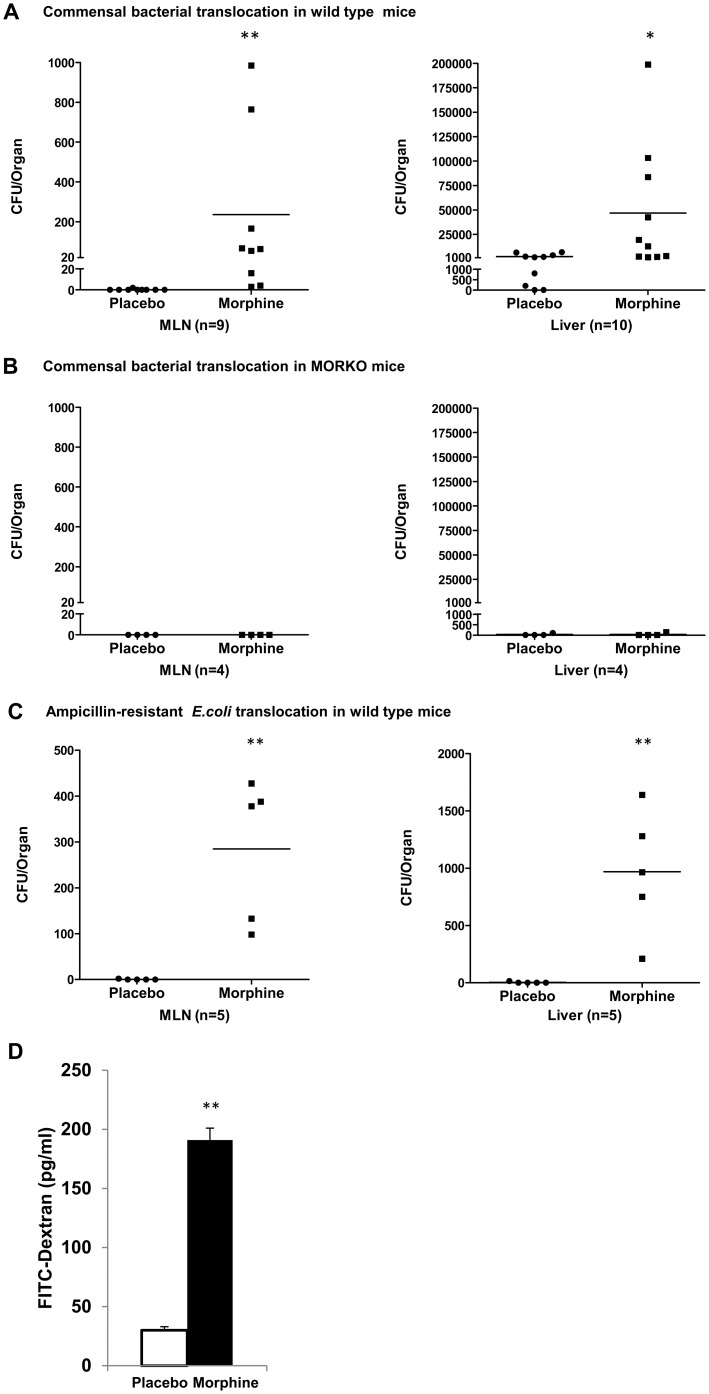
Chronic morphine compromises barrier function of gut epithelium and promotes bacterial translocation. Wild type (A) and MORKO (B) mice were treated with 75 mg morphine pellets for 24 hours, MLN and liver homogenates were cultured on blood agar plate overnight. Bacterial colonies were quantified and described as colony forming units (CFU). (C) WT mice were gavaged with ampicillin -resistant E. coli after morphine treatment, and the number of E. coli in MLN and liver were quantified using an LB agar plate containing ampicillin. (D) The permeability of gut epithelium increased after morphine treatment as determined by measuring the whole blood FITC-dextran concentration.– Median of CFU; (A) to (C)** p<0.01 *P<0.05 by Mann–Whitney test. (D) **P<0.01 by Student's t-test.

To determine whether chronic morphine treatment modulates bacterial dissemination, we determined spontaneous gut bacterial translocation following morphine treatment. B6129PF2 wild type mice were implanted with 75 mg morphine pellet or placebo pellet subcutaneously. Mesenteric lymph node (MLN) (n = 9) and liver (n = 10) suspensions were collected after 24 hours, cultured on blood agar plates (BD Biosciences) overnight and the colony forming units (CFUs) were quantified. Placebo-implanted mice showed no colonies growing on the plates, indicating no bacterial translocation. Conversely, mice receiving morphine revealed an increased number of CFUs, indicating bacterial dissemination to MLN and liver following 24 hours of morphine treatment (Figure 1A). At 48 hours, morphine-induced bacterial translocation into liver and MLN persisted (Figure S1). To determine the role of μ-opioid receptors (MOR) in morphine modulation of bacterial translocation, we implanted MOR knockout (MORKO) mice with morphine pellets, as described above. Morphine-induced bacterial translocation was completely abolished in MORKO mice (Figure 1B), suggesting that MOR mediated morphine's effects on bacterial translocation. To further confirm that the disseminated bacteria were from the gut lumen rather than opportunistic infections, we gavaged WT mice with ampicillin-resistant *E.coli* and quantified bacterial translocation with Lysogeny broth (LB) plates containing ampicillin. Morphine-treated mice showed ampicillin-resistant *E.coli* dissemination into MLN and liver (Figure 1C), indicating that morphine treatment promotes bacterial translocation of commensal bacteria from the gut lumen. In addition, morphine treatment promoted fluorescein isothiocyanate (FITC)-conjugated dextran translocation from gut lumen to blood (Figure 1D), suggesting that morphine increased the permeability of the gut epithelium. Serotyping of the disseminated bacteria (Veterinary Diagnostic Laboratory, University of Minnesota) revealed a prevalence of *Staphylococcus, Enterococcus, and Bacillus sp.*, which are commensal bacteria in the gut lumen.

### Chronic morphine induces inflammation and disrupts organization of tight junction proteins between epithelial cells in small intestine

**Figure 2:**
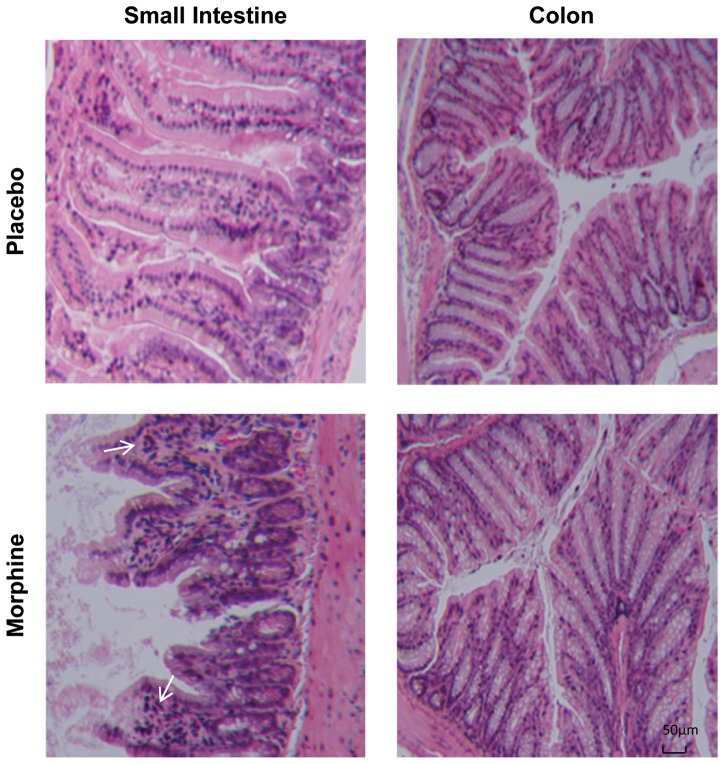
Chronic morphine induces inflammation in small intestine. Representative hematoxylin and eosin (H&E)-stained sections from the small intestine and colon of placebo- and morphine-treated WT mice. White arrow indicates inflammatory cell infiltration.

**Figure 3:**
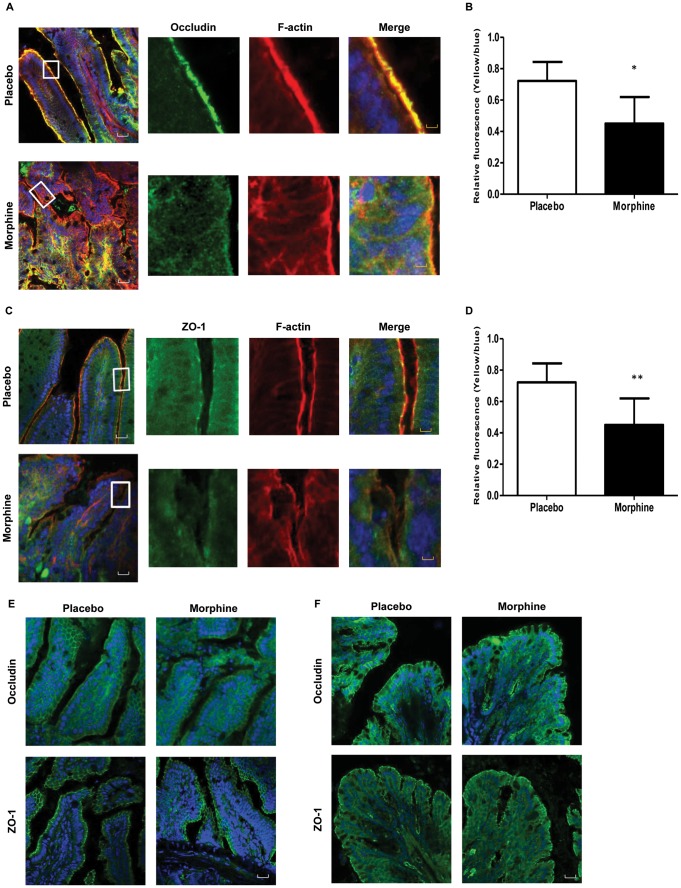
Chronic morphine disrupts tight junction organization between small intestinal epithelial cells. (A) Occludin organization in small intestine of WT mice. (C) ZO-1 organization in small intestine of WT mice. Quantification of co-localization of occludin (B) or ZO-1 (D) with F-actin are showed as relative intensity of yellow fluorescence normalized to blue fluorescence (DAPI) (E) Occludin and ZO-1 organization in small intestine of MORKO mice. (F) Occludin and ZO-1 organization of colon in WT mice. WT and MORKO mice were treated with 75 mg morphine pellet for 24 hours. The same parts of small intestines and colons were excised and fixed. Images were analyzed by confocal scanning microscope. (n = 5) Scale bar: white 50 µm; yellow 10 µm * P<0.05, **P<0.01 by Student's t-test.

To investigate the effects of morphine on the morphology of the intestinal epithelium, small intestine and colon were excised and fixed in a formalin solution for hematoxylin and eosin (H&E) staining. Histological analysis showed injured epithelium and increased inflammatory infiltrates in small intestinal villi of morphine-treated mice (Figure 2). In contrast, no morphological change was observed in the colon of morphine-treated mice, suggesting a differential effect by morphine on small intestinal and colonic epithelium. Our findings of morphine-induced microbial translocation and barrier compromise in the small intestine of mice prompted us to study the tight-junction organization of the intestinal epithelium. Wild-type mice were implanted with placebo or 75 mg morphine pellet for 24 hours. Then parts of the small intestine were excised, frozen and 5 µm sections were cut. The sections were stained for occludin and zona occludens 1 (ZO-1), two proteins integral to the formation of epithelial tight-junction. In placebo treated mice, the trans-membrane protein occludin localized to the apical side of epithelium with a continuous and intact organization (Figure 3A). Images showed that occludin co-localized with the well-organized F-actin on the membrane of epithelial cells of placebo-treated mice (Figure 3A). In contrast, morphine treated mice showed disrupted localization of occludin, suggesting impaired recruitment of the protein to the membrane (Figure 3A). Similar to occludin, the paracellular tight junction protein ZO-1 also localized with F-actin on the apical side of the membrane in placebo-treated mice, and its organization was seen to be disrupted following 24 hours of morphine treatment (Figure 3C). Morphine treatment did not change the expression levels of occludin or ZO-1 (Figure S2), suggesting that morphine modulated the distribution of tight junction proteins, resulting in increased intestinal permeability. Quantification of yellow fluorescence (indicating the co-localization of red and green) also showed significant reduction in the co-localization of tight junction and F-actin in morphine-treated mice (Figure 3B and D). In MORKO mice, consistent with our bacterial translocation data, morphine did not have any effect on occludin and ZO-1 organization in the small intestine, indicating that morphine's effect on intestinal tight junction were mediated by MOR (Figure 3E). Interestingly, morphine did not have an effect on either occludin or ZO-1 organization in the colonic epithelium, where both placebo- and morphine-treated mice showed intact and continuous localization of occludin and ZO-1 (Figure 3F). This finding suggests the differential regulation of barrier functions in different compartments of the gastrointestinal epithelium.

### Morphine treatment up-regulates TLR expression in epithelial cells of small intestine

**Figure 4:**
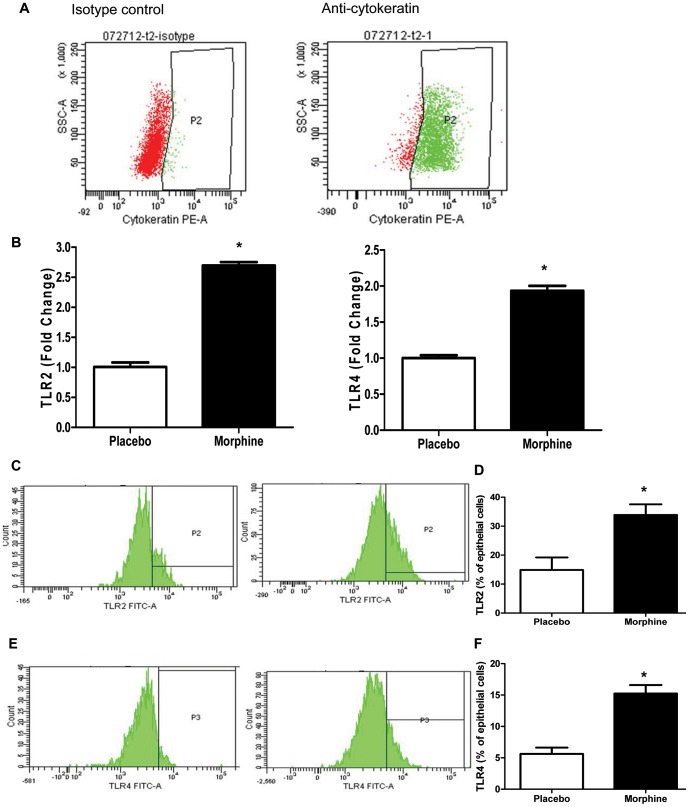
Morphine treatment upregulates TLR expression in small intestinal epithelial cells. (A) Isolated cells were fixed using eBioscience Fixation and Permeabilization Kit and then incubated with anti-cytokeratin antibody or isotype control. Cytokeratin positive cells were gated in P2 according to isotype control. (B) Real-time PCR analysis of mRNA levels of TLR2 and TLR4 in epithelial cells of small intestine after 24 hour morphine treatment. (C) and (E) Representative expression of TLR2 and TLR4 in epithelial cells of small intestine after 24 hour morphine treatment from 3-time experiments. (D) and (F) Frequencies of TLR2 and TLR4 positive cells within cytokeratin positive cells. * P<0.05 by Student's t-test.

As we have discussed previously, there is a clear correlation between TLR activation and tight junction disruption in intestinal mucosa, consistent with instances recently described in the literature [30], [31]. To determine whether TLR expression on gut epithelial cells is one mechanism by which morphine modulates barrier function, we implanted mice with placebo or morphine pellets for 24 hours and isolated epithelial cells from the small intestines as described previously [25]. Total RNA was isolated from these cells and processed for qPCR. For flow cytometery, the isolated cells were gated by cytokeratin as an epithelial marker [32] (Figure 4A).Results showed 24 hours of morphine treatment up-regulated both mRNA (Figure 4B) and protein levels (Figure 4C–F) of TLR2 and TLR4. In addition, the messenger RNA levels of TLR2 and TLR4 in colonic epithelial cells following morphine treatment was determined by gel-based PCR (). The results showed that neither TLR2 nor TLR4 was significantly up-regulated by morphine in the colonic epithelium in contrast to the observation in the small intestinal epithelium.

### Morphine-induced bacterial translocation is attenuated in TLR2/TLR4 knockout mice

**Figure 5:**
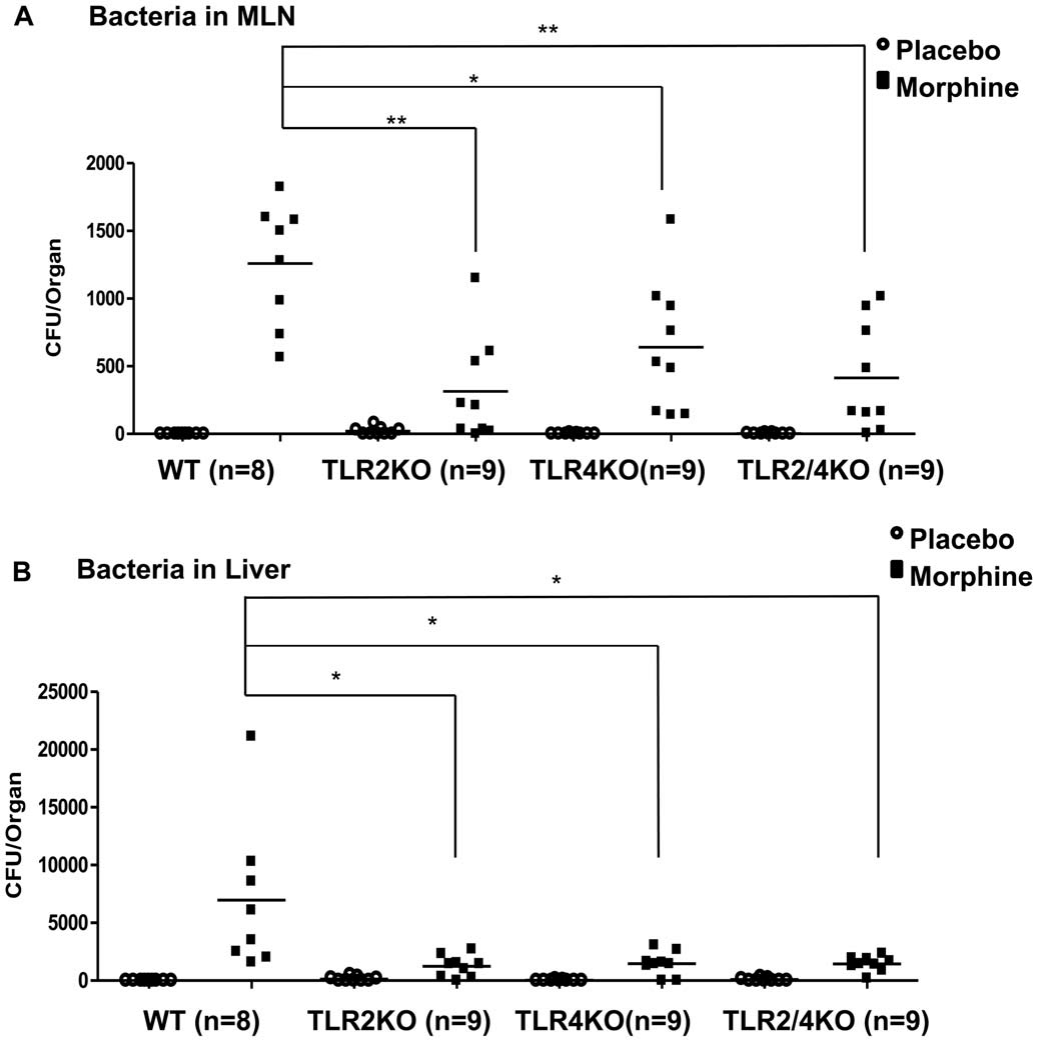
Morphine-induced bacterial translocation is attenuated in TLR2/TLR4 knockout mice. WT, TLR2 knockout, TLR4 knockout, and TLR2/4 double knockout mice were implanted with 75 mg morphine pellet for 24 hours; MLN(A), liver (B) were cultured on blood agar plates overnight. Bacterial colonies were quantified and described as CFU. – Mean of CFU *P<0.05, **P<0.01 by ANOVA one-way analysis, followed by Bonferroni post-test (n = 9).

To further determine roles of TLR2 and TLR4 in morphine-induced bacterial translocation, we implanted C57BL/6 J WT (n = 9), TLR2 knockout (n = 9), TLR4 knockout (n = 9), and TLR2/4double knockout (n = 9) mice with morphine pellets to determine bacterial load in MLN and liver as described previously. Placebo-treated TLR 2, 4 KO mice showed very low basal levels of bacterial load in MLN and liver. Morphine-treated WT mice still showed significant bacterial translocation to MLN and liver. In contrast, morphine-treated TLR2, 4 knockout mice showed lower bacterial translocation into MLN and liver than did WT mice (Figure 5) although TLRKO did not show any effects on morphine-induced constipation, suggesting that constipation is not the only dominant factor causing bacterial translocation following morphine treatment and other TLR-dependent mechanisms also contribute to the process of TJ disorganization and barrier dysfunction (Figure S3). These findings indicated that both TLR2 and TLR4 are involved in morphine modulation of intestinal barrier function.

### TLR2/TLR4 knockout protects tight junction organization from morphine-induced disruption

**Figure 6:**
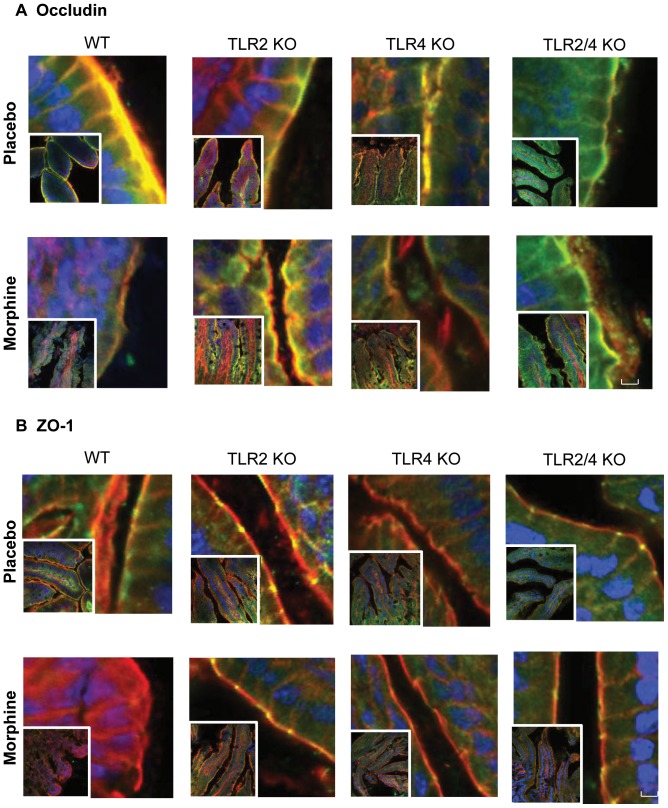
TLR2/TLR4 knockout protects tight junction organization from morphine-induced disruption. (A) Occludin organization in small intestine of WT and TLRKO mice. (B) ZO-1 organization in small intestine of WT and TLRKO mice. WT, TLR2 knockout, TLR4 knockout, and TLR2/4 double knockout mice were implanted with 75 mg morphine pellet for 24 hours. The similar parts of small intestines were excised and fixed. Images were analyzed by confocal scanning microscope. (n = 5) Scale bar: 10 µm.

To further determine the role of TLRs in morphine's modulation of intestinal tight junction proteins, we isolated the small intestine from WT, TLR2 knockout, TLR4 knockout, and TLR2/4 double knockout mice to assess the organization of tight junction proteins, as described previously. In TLR2KO and TLR2/4KO mice, the occludin and ZO-1 staining were continuous and intact following morphine treatment (Figure 6A and 6B). In TLR4KO mice, some degree of tight junction disruption was observed following morphine treatment; however, the disruption was not as dramatic as that observed with morphine treatment in WT mice, suggesting a dominant role of TLR2 in morphine modulation of intestinal tight junction organization, which was consistent with our *in vitro* study: small intestinal cell IEC-6 and colonic epithelial cell CMT-93 were stained for tight junction proteins ZO-1(Figure S4). LPS and LTA but not morphine induced ZO-1 internalization. And morphine enhanced LTA's effects on IEC-6 cells, further validating that TLR2 plays a more dominant role in TJ modulation in gut epithelial cells following morphine treatment. In contrast, neither LPS nor LTA showed any effect on TJ distribution in colonic CMT-93 cells, consistent with our *in vivo* data (Figure S4).

### TLR signaling modulates intestinal tight junction organization in a MLCK-dependent manner

**Figure 7:**
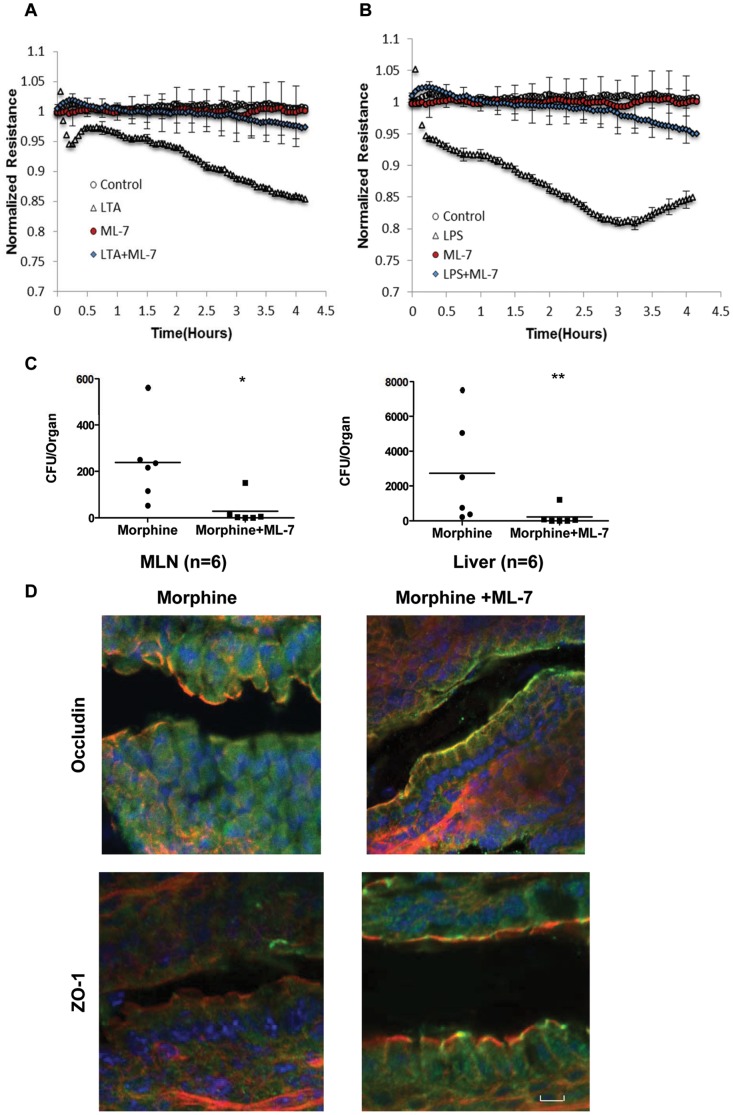
TLR signaling modulates intestinal tight junction organization in a MLCK-dependent manner. (A) Effects of LTA on TER of IEC-6 cells are blocked by MLCK inhibition. (B) Effects of LPS on TER of IEC-6 cells are blocked by MLCK inhibition. (C) Bacterial translocation to MLN and liver are blocked by MLCK inhibition. ** p<0.01 *P<0.05 by Mann–Whitney test. (D) MLCK inhibition protects tight junction organization following morphine treatment. (n = 6) Scale bar: 10 µm.

Since our data (Figure S2) show that TLR ligands have no effect on tight junction protein expression levels, the increased permeability of epithelial cells by TLR activation may involve post-translation mechanisms. Recent studies showed that myosin light chain kinase (MLCK) regulates the contraction of tight junctions by phosphorylating myosin light chains [24], [33], [34]. Activation of MLCK induces phosphorylation of the myosin light chains, resulting in the contraction of cytoskeleton proteins such as F-actin and thus inducing the internalization of associated tight junction proteins such as occludin and ZO-1. To determine whether MLCK is responsible, we determined the barrier function of IEC-6 cells by electrical cell impedance sensing (ECIS) arrays. The cells were grown to confluence in ECIS arrays, and the trans-epithelial resistance (TER) values were measured to test whether morphine would affect epithelial barrier integrity. The baseline TER of each experiment was normalized to 1.0 to enable comparison and statistical analysis of TER changes over time following different treatments. IEC-6 cells were treated with MLCK inhibitor ML-7, and the TER values were measured in the presence of LTA (Figure 7A) and LPS (Figure 7B). Inhibition of MLCK restored the TER values to the control levels, indicating that the effects of TLR agonists on epithelial cells are dependent on MLCK. To further validate the role of MLCK in tight junction modulation, WT mice were injected with 2 mg of ML-7/kg body weight prior to morphine treatment as described previously [24]. ML-7 inhibited morphine-induced bacterial translocation to MLN and liver (Figure 7C), and protected occludin and ZO-1 organization from morphine-induced disruption (Figure 7D), although it did not block constipation caused by morphine treatment (Figure S3).

## Discussion

In the current study, we show that morphine mediated signaling by μ-opioid receptors 1) induced bacterial dissemination into MLN and liver of WT mice; 2) compromised intestinal barrier function; and 3) disrupted tight junction organization in gut epithelial cells through a TLR- dependent mechanism.

Our studies show significant bacterial translocation to the mesenteric lymph node and liver of WT mice that are morphine treated (Figure 1A and Figure S1). Over the past two decades, a large amount of studies have been conducted to investigate the effects of morphine on bacterial translocation and intestinal permeability using various rodent models. Consistently these studies demonstrate that morphine alters intestinal transit and promote bacterial translocation in rodents [35], [36] although in one study morphine only in the presence of TNF was able to increase intestinal permeability [37]. Bacterial translocation was not measured in these studies [37]. It is not clear why there is a discrepancy between this study and the majority of other studies but the differences in the results may be attributed to differences in the doses of morphine used, the route of administration or the sensitivity of the permeability experiments. However, most recent studies clearly establish that morphine treatment in doses that are clinically relevant results in bacterial translocation in both rats and mice [7], [36]. In addition, we rule out the possibility that the bacteria detected in liver and lymph node is not a consequence of opportunistic infections due to suppressed immune function by morphine by measuring ampicillin-resistant E. coli and FITC-conjugated dextran translocation (Figure 1C and 1D), validating that the observed bacterial translocation is a consequence of disrupted intestinal barrier function following chronic morphine treatment. We further show that morphine's effects were abolished in the MOR knockout mice (Figure 1B), indicating that morphine's modulatory effect on intestinal barrier function were mediated by MOR.

We then demonstrated through morphological evaluation of the gut that morphine potentiated inflammation in small intestine. Histological analysis showed injured epithelium and increased inflammatory infiltrates in the villi of the small intestines in morphine-treated mice (Figure 2), which was usually associated with disrupted intestinal barrier function [16]. Interestingly, we failed to observe any effect of morphine on colonic epithelium (Figure 2), suggesting a differential effects of morphine on small intestinal and colonic derived epithelium, despite the observation that MOR expression is similar in the colon and in the small intestine (Figure S6). These observations are consistent with the recent studies by Ross *et al* [38] where it was demonstrated that tolerance to morphine is differentially regulated in the ileum versus the colon. Although, in this study, the cellular basis for the differential expression of morphine tolerance in the ileum versus the colon was not defined, it is conceivable that signaling downstream of MOR activation may contribute to the differential effect.

Our studies also demonstrated that the organization of tight junction proteins in small intestines were disrupted following morphine treatment (Figure 3A to D), suggesting paracellular translocation of bacteria from the gut lumen. Tight junction proteins have been shown to seal the gap between gut epithelial cells and play an important role in preventing potential pathogen invasion [16]. Interestingly, morphine did not affect tight junction proteins' expression levels in intestinal epithelial cells (Figure S2), implying that it is their **distribution** that is involved in modulating intestinal permeability. To understand the cellular mechanism underlying tight junction modulation by morphine, we used IEC-6 cells as an *in vitro* model and determined its tight junction distribution following morphine treatment. To our surprise, morphine alone showed no effect on tight junction of epithelial cells. However, we observed that TLR2 and TLR4 ligands disrupted the tight junction organization of monolayers formed by small intestinal epithelial cells (IEC-6). Morphine modulated TJ organization of IEC-6 cells only in the presence of TLR2 ligand, suggesting that morphine's effects were mediated by TLRs. On the other hand, neither morphine nor TLR ligands showed any effect on barrier function of colonic epithelial cells (Figure S4), implying differential regulation of TJ in the ileum and colon by TLRS.

Historically, many studies have investigated the role of TLRs in modulating tight junctions in various epithelial cells: invasive bacterial pathogens *S. pneumoniae* and *H. influenzae* were observed to translocate across the epithelium through TLR-dependent down-regulation of tight junction components [39]. LPS also has been reported to disrupt tight junction of cholangiocytes–the epithelial cells of the bile duct–by a TLR4-dependent mechanism [30]. Our *in vivo* studies support the role of TLRs in tight junction modulation in gut epithelial cells. Protein levels of TLR2 and TLR4 were increased in small intestine following morphine treatment (Figure 4). Bacterial translocation and tight junction disruption were significantly attenuated in TLR2KO, TLR4KO, and TLR2/4 double knockout mice following morphine treatment (Figure 5 and 6), demonstrating that both TLR2 and TLR4 contribute to morphine-induced intestinal barrier disruption. Interestingly, TLR4 signaling was not involved in morphine modulation of epithelial barrier function in IEC-6 cells (Figure S3), which was contradictory to our *in vivo* study, where we show significant protection of tight junction from morphine-induced disruption in TLR4 knockout. These results suggest that activation of TLR4 in other cell types and not on the epithelial cells may play a more dominant role in morphine modulation of epithelial barrier function. TLR4 has been shown to play an important role in cytokine production in gut associated lymphoid tissue (GALT), which plays crucial roles in maintaining intact intestinal barrier function and defense against potential pathogen invasion [40]. We postulate that TLR4 activation in the GALT, but not in epithelial cells, is involved in gut barrier modulation. In support of this hypothesis, it has been demonstrated that abnormal pro-inflammatory cytokine production induced by translocated bacteria causes disruption of tight junction proteins in gut epithelium [41]. This feed-forward vicious cycle contributes to serious gut inflammatory disease and even sepsis. Therefore, it is conceivable that other factors activated by TLR4 may play a role in disrupting intestinal barrier function by modulating pro-inflammatory cytokines TNF-alpha and IL-6 [42].

In addition, both *in vitro* and *in vivo* studies demonstrated that the distribution of tight junction was modulated by myosin light chain kinase (MLCK). MLCK inhibition completely blocked LTA- and LPS- induced barrier dysfunction in IEC-6 cells and morphine-induced bacterial dissemination in mice (Figure 7), which confirmed that the impaired barrier function of epithelial cells following TLR activation is due to MLCK-induced redistribution of tight junction proteins rather than decreased tight junction protein expression levels.

**Figure 8:**
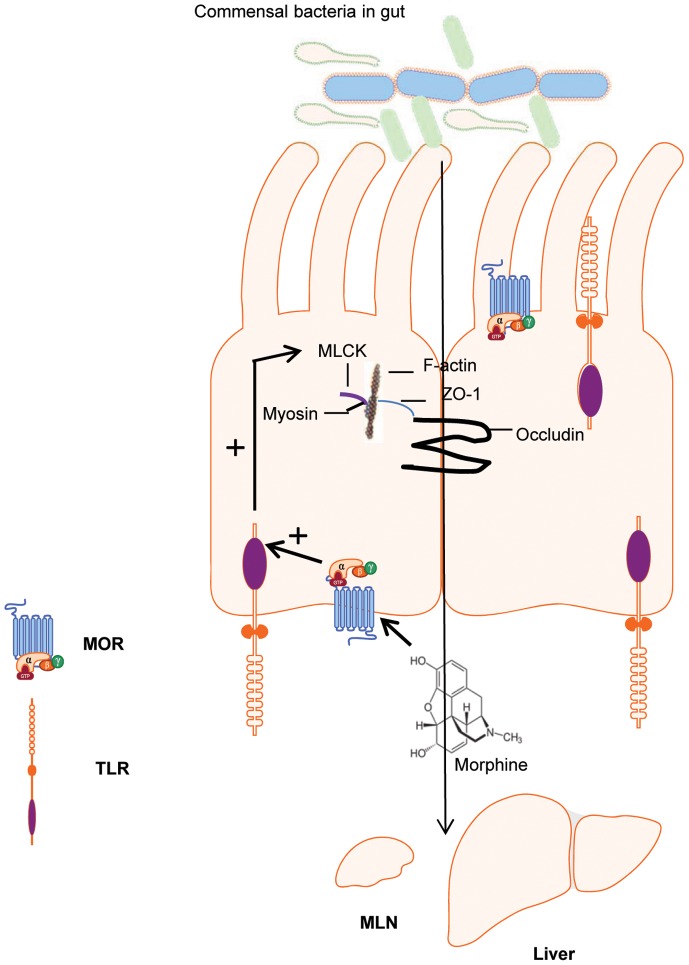
Model of morphine-induced disruption of gut epithelial barrier function. Morphine treatments up-regulate TLR expression levels in small intestinal epithelial cells. Activated TLR signaling induces tight junction disruption between epithelial cells and increases gut permeability, resulting in increased bacterial translocation.

In summary, our studies demonstrate that morphine treatment up-regulates TLR expression levels in small intestinal epithelial cells and sensitized small intestinal epithelial cells to TLR stimulation, which induced disruption of tight junctions between epithelial cells, increased gut permeability, and resulted in increased bacterial translocation and inflammation in the small intestine (Figure 8). In contrast, colonic epithelium did not show any response to morphine treatment, suggesting differential effects of morphine on small intestinal and colonic barrier function. Currently, opiates are among the most prescribed drugs for pain management. However, they induce multiple adverse gastrointestinal symptoms including dysfunction of the gut immune system, which may lead to a higher risk of gut bacterial infection as well as faster progression of infectious diseases such as sepsis. These adverse effects seriously affect patients' quality of life and limit the prolonged use of opiates for pain management. These studies contribute to the urgent need to understand the mechanism through which morphine modulates intestinal barrier function, enhancing our ability to develop novel strategies for treating or preventing gut bacterial infection or sepsis in opiate-using or -abusing populations.

## Supporting Information

Figure S1 **48 hours of Morphine treatment promotes bacterial translocation in wild type mice.** Wild type mice were treated with 75 mg morphine pellet for 48 hours, mesenteric lymph node and liver were isolated, homogenized and cultured on blood agar plate overnight. Bacterial colonies were quantified and described as colony forming units (CFU) (n = 3).
(PDF)

Figure S2 **Occludin and ZO-1 expression of total small intestinal epithelial cells.** Small intestinal epithelial cells were isolated from placebo and morphine-treated mice and lysed with RIPA buffer. The sample was used for WB. Figure B is the quantification of 3-time experiments.
(PDF)

Figure S3 **Morphine induces constipation in mice.** Pictures of intestines from placebo- and morphine-treated WT, TLR2KO, TLR4KO, TLR2/4KO mice in absence or presence of ML-7.
(PDF)

Figure S4 **Morphine's effects on tight junction of IEC-6 and CMT-93 cells.** IEC-6 and CMT-93 Cells were fixed and incubated with anti-zo-1 antibody, followed by FITC-labeled secondary antibody. Magnification ×600.
(PDF)

Figure S5 **Morphine's effects on TLR expression in small intestinal and colonic epithelial cells.** Gel-based PCR analysis of mRNA levels of TLR2 and TLR4 in epithelial cells of small intestinal and colonic epithelial cells after morphine treatment. P: Placebo M: Morphine.
(PDF)

Figure S6 **MOR expression in small intestinal and colonic epithelial cells.** Gel-based PCR analysis of mRNA levels of MOR in epithelial cells of small intestinal and colonic epithelial cells. SI: Small intestine; C: Colon.
(PDF)
