# Supplementary figures and images for: Morphine Induces Bacterial Translocation in Mice by Compromising Intestinal Barrier Function in a TLR-Dependent Manner
Source: PLoS One. 2013 Jan 18;8(1):e54040. doi: 10.1371/journal.pone.0054040 (PMC3548814; doi:10.1371/journal.pone.0054040)

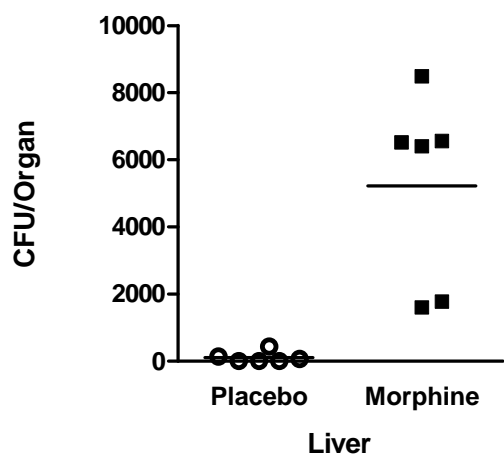

Supplement: Figure S1 — 48 hours of Morphine treatment promotes bacterial translocation in wild type mice. Wild type mice were treated with 75 mg morphine pellet for 48 hours, mesenteric lymph node and liver were isolated, homogenized and cultured on blood agar plate overnight. Bacterial colonies were quantified and described as colony forming units (CFU) (n = 3). (PDF) [file pone.0054040.s001.pdf]

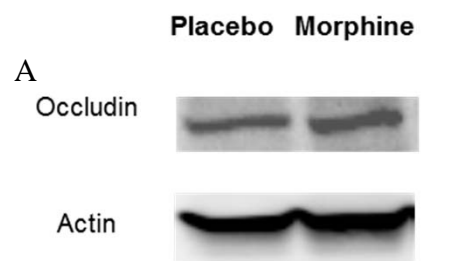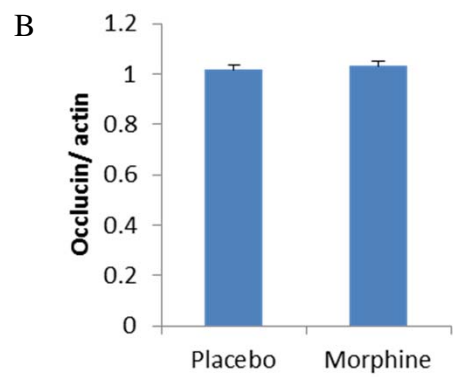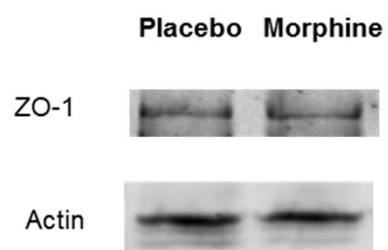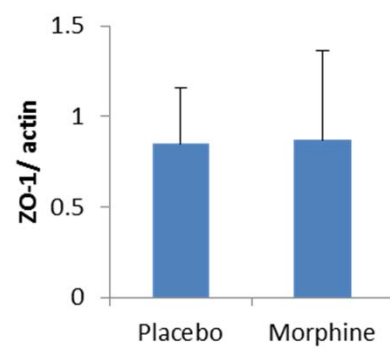

Supplement: Figure S2 — Occludin and ZO-1 expression of total small intestinal epithelial cells. Small intestinal epithelial cells were isolated from placebo and morphine-treated mice and lysed with RIPA buffer. The sample was used for WB. Figure B is the quantification of 3-time experiments. (PDF) [file pone.0054040.s002.pdf]

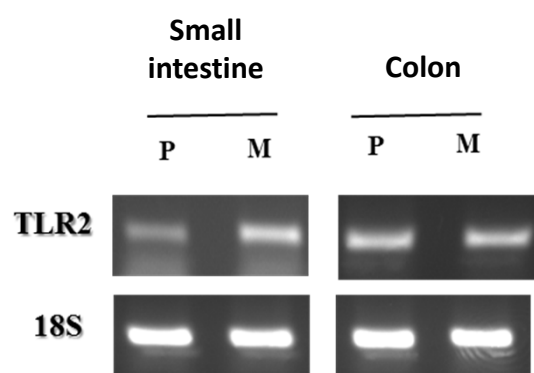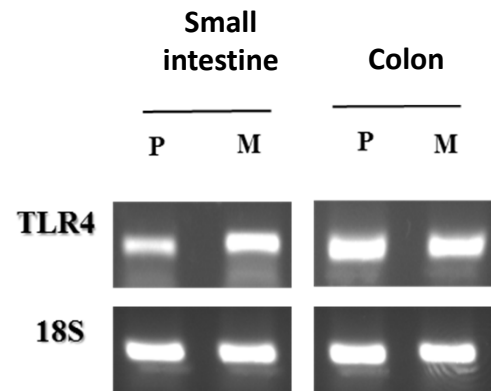

Supplement: Figure S5 — Morphine's effects on TLR expression in small intestinal and colonic epithelial cells. Gel-based PCR analysis of mRNA levels of TLR2 and TLR4 in epithelial cells of small intestinal and colonic epithelial cells after morphine treatment. P: Placebo M: Morphine. (PDF) [file pone.0054040.s005.pdf]

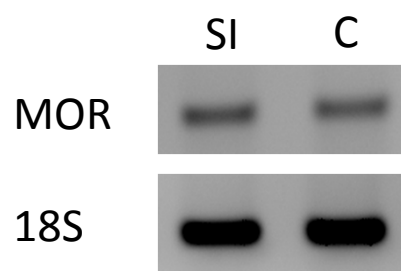

Supplement: Figure S6 — MOR expression in small intestinal and colonic epithelial cells. Gel-based PCR analysis of mRNA levels of MOR in epithelial cells of small intestinal and colonic epithelial cells. SI: Small intestine; C: Colon. (PDF) [file pone.0054040.s006.pdf]
