# Supplementary material for: Morphine Induces Bacterial Translocation in Mice by Compromising Intestinal Barrier Function in a TLR-Dependent Manner
Source: PLoS One. 2013 Jan 18;8(1):e54040. doi: 10.1371/journal.pone.0054040 (PMC3548814; doi:10.1371/journal.pone.0054040)

WT: Placebo

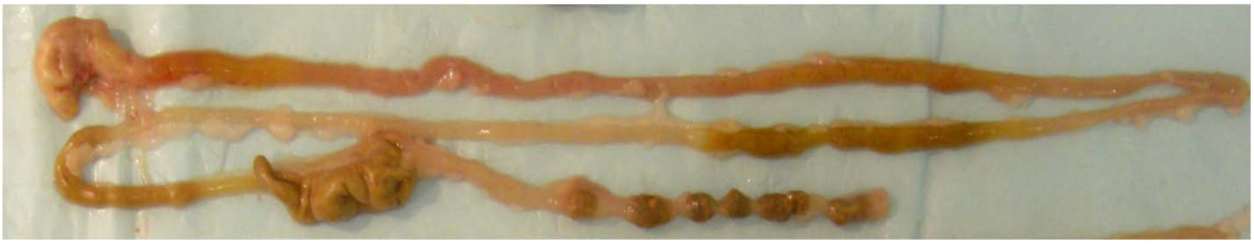

Morphine

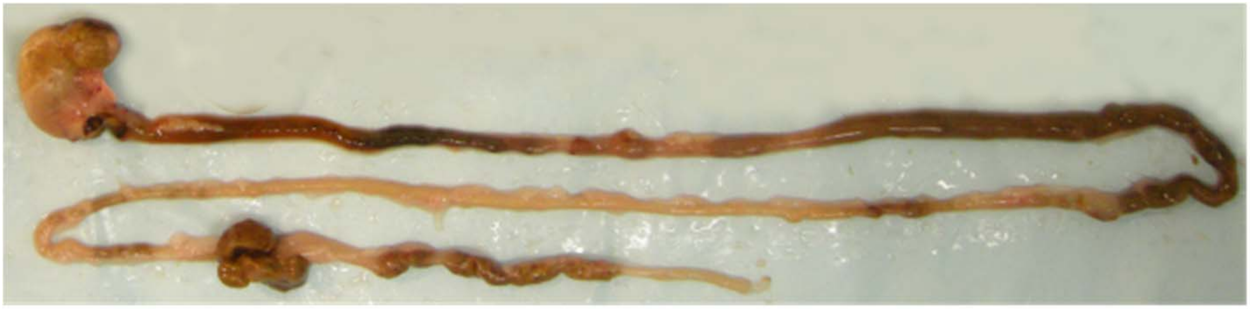

TLR2KO: Placebo

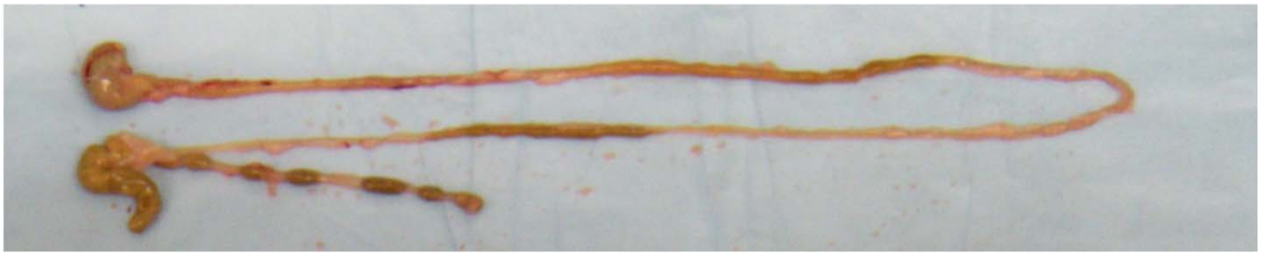

Morphine

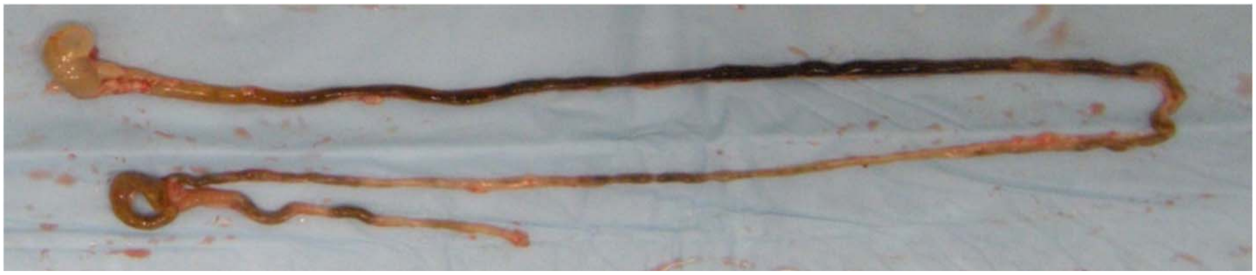

TLR4KO: Placebo

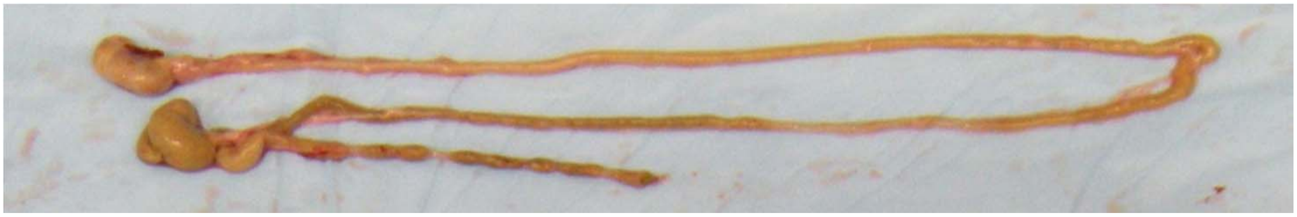

Morphine

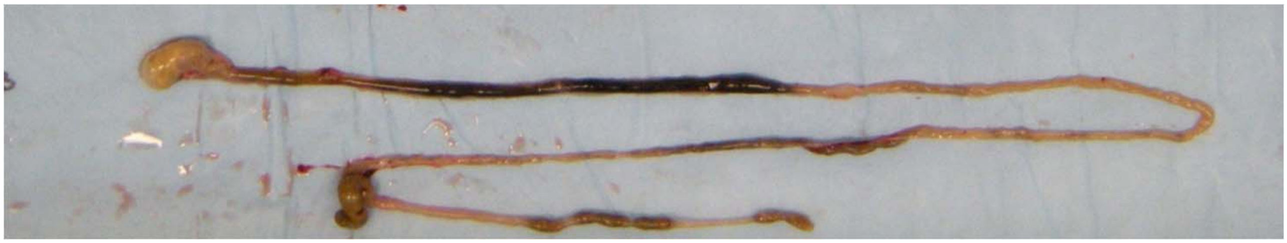

TLR2/4KO: Placebo

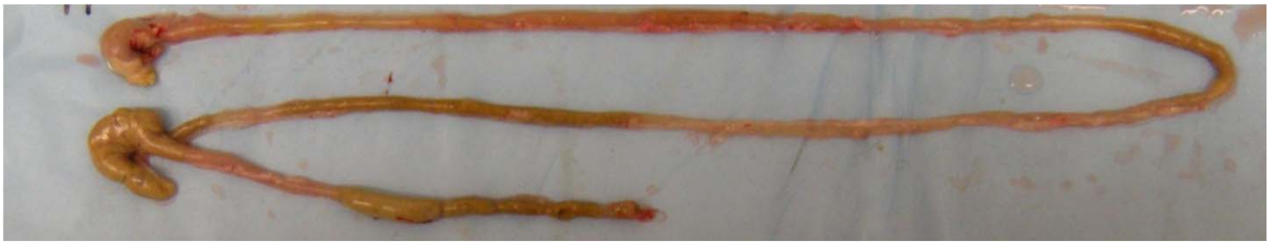

Morphine

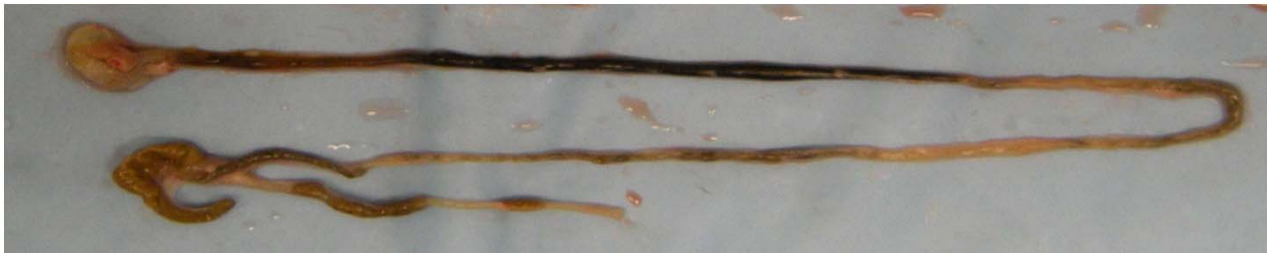

WT: Morphine +Saline

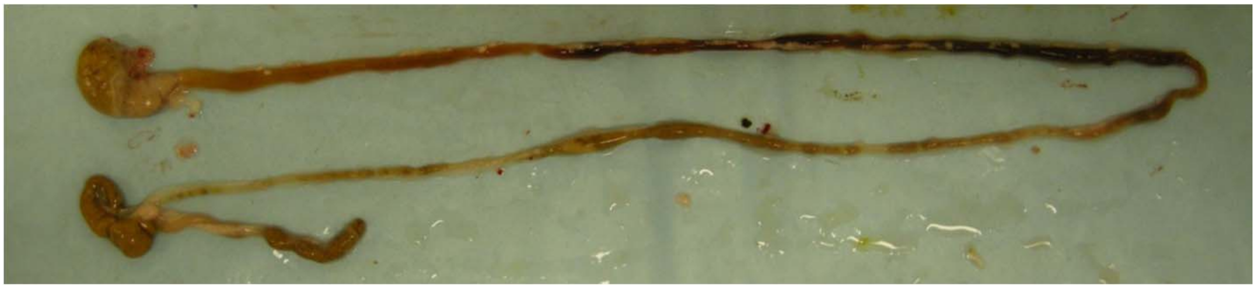

Morphine +ML-7

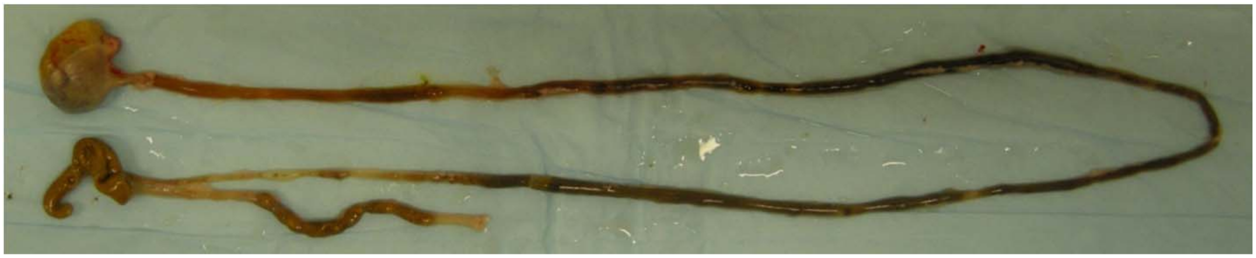

Supplement: Figure S3 — Morphine induces constipation in mice. Pictures of intestines from placebo- and morphine-treated WT, TLR2KO, TLR4KO, TLR2/4KO mice in absence or presence of ML-7. (PDF) [file pone.0054040.s003.pdf]
