# Supplementary material for: Morphine Induces Bacterial Translocation in Mice by Compromising Intestinal Barrier Function in a TLR-Dependent Manner
Source: PLoS One. 2013 Jan 18;8(1):e54040. doi: 10.1371/journal.pone.0054040 (PMC3548814; doi:10.1371/journal.pone.0054040)

A: IEC-6 Cells

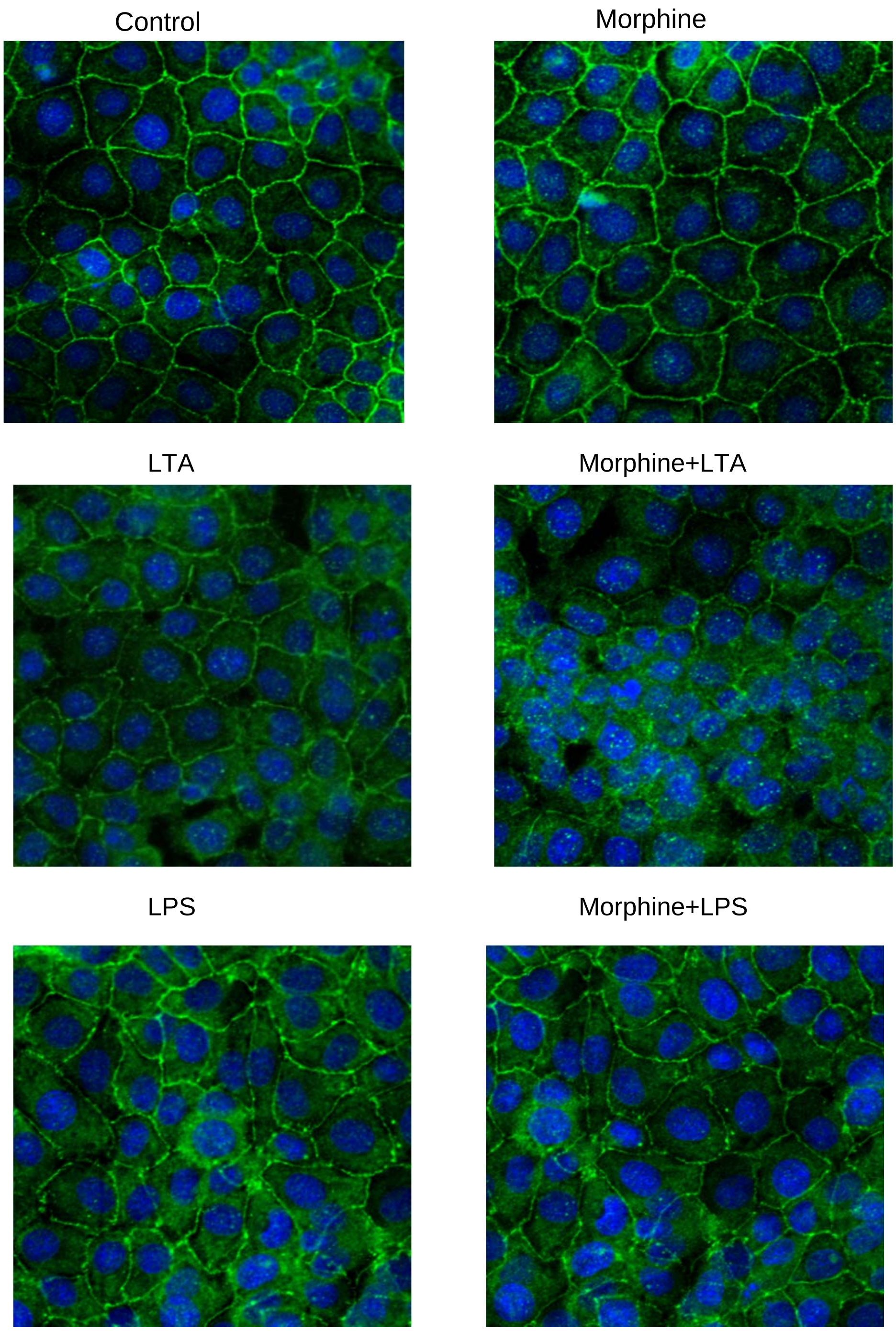

B: CMT-93Cells

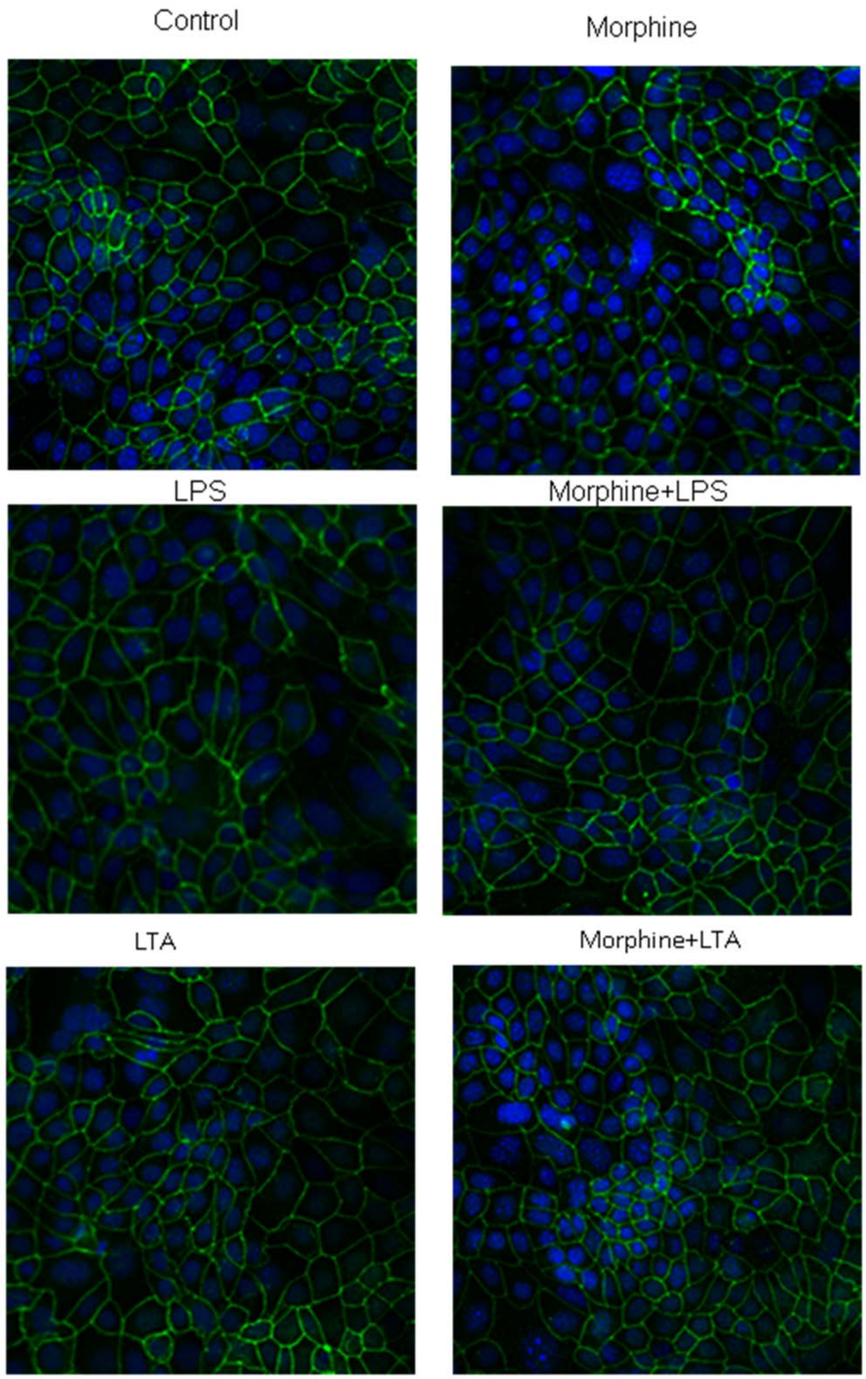

Supplement: Figure S4 — Morphine's effects on tight junction of IEC-6 and CMT-93 cells. IEC-6 and CMT-93 Cells were fixed and incubated with anti-zo-1 antibody, followed by FITC-labeled secondary antibody. Magnification ×600. (PDF) [file pone.0054040.s004.pdf]
